# Evaluating a multimodal, clinical and work-directed intervention (RTW-PIA) to support sustainable return to work among employees with mental disorders: study protocol of a multicentre, randomised controlled trial

**DOI:** 10.1186/s12888-023-04753-5

**Published:** 2023-05-30

**Authors:** Fiona Starke, Alexandra Sikora, Ralf Stegmann, Leonie Knebel, Claudia Buntrock, Angelique de Rijk, Inge Houkes, Gregor R. Szycik, Hans-Peter Unger, Jan Ole Schumacher, Heiko Stark, Iris Hauth, Christian Holzapfel, Anna Borgolte, Carlotta Schneller, Sarah-Louise Unterschemmann, Wiebke Paetow, Anna Lena Jung, Matthias Berking, Johannes Zimmermann, Uta Wegewitz

**Affiliations:** 1 Division 3 Work and Health, Unit 3.5 Evidence-based Occupational Health, Workplace Health Management, Federal Institute for Occupational Safety and Health (BAuA), Nöldnerstr. 40-42, Berlin, 10317 Germany; 2 Department of Social Medicine, Faculty of Health, Medicine and Life Sciences, CAPHRI Care and Public Health Research Institute, Maastricht University, P.O. Box 616, Maastricht, 6200 MD The Netherlands; 3 Institute of Social Medicine and Health Systems Research, Faculty of Medicine, Otto-Von-Guericke University Magdeburg (OVGU), Leipziger Str. 44, 39120 Magdeburg, Germany; 4 Department of Clinical Psychology and Psychotherapy, Institute of Psychology, Friedrich-Alexander-Universität Erlangen-Nürnberg (FAU), Nägelsbachstr. 25a, 91052 Erlangen, Germany; 5 Department of Psychiatry, Social Psychiatry and Psychotherapy, Hanover Medical School, Podbielskistr. 162, Hanover, OE7110 Germany; 6 Department of Psychiatry, Psychotherapy and Psychosomatics, Centre for Mental Health, Asklepios Clinic Harburg, Eißendorfer Pferdeweg 52, Hamburg, 21075 Germany; 7 Department of Psychiatry, Burghof-Clinic, Ritterstr. 19, 31737 Rinteln, Germany; 8 Department of Psychiatry, Psychotherapy and Psychosomatics, Alexian St. Joseph-Hospital Berlin-Weissensee, Gartenstr. 1, 13088 Berlin, Germany; 9 Clinic Wittgenstein, Sählingsstr. 60, 57319 Bad Berleburg, Germany; 10 Department of Psychology, University of Kassel, Holländische Str. 36-38, 34127 Kassel, Germany

**Keywords:** Return to work, Mental disorders, Relapse prevention, RCT, Sickness absence, Multimodal Intervention, Sustainability, SRTW, Protocol

## Abstract

**Background:**

Mental disorders (MDs) are one of the leading causes for workforce sickness absence and disability worldwide. The burden, costs and challenges are enormous for the individuals concerned, employers and society at large. Although most MDs are characterised by a high risk of relapse after treatment or by chronic courses, interventions that link medical-psychotherapeutic approaches with work-directed components to facilitate a sustainable return to work (RTW) are rare. This protocol describes the design of a study to evaluate the (cost-)effectiveness and implementation process of a multimodal, clinical and work-directed intervention, called RTW-PIA, aimed at employees with MDs to achieve sustainable RTW in Germany.

**Methods:**

The study consists of an effectiveness, a health-economic and a process evaluation, designed as a two-armed, multicentre, randomised controlled trial, conducted in German psychiatric outpatient clinics. Sick-listed employees with MDs will receive either the 18-month RTW-PIA treatment in conjunction with care as usual, or care as usual only. RTW-PIA consists of a face-to-face individual RTW support, RTW aftercare group meetings, and web-based aftercare. Assessments will be conducted at baseline and 6, 12, 18 and 24 months after completion of baseline survey. The primary outcome is the employees´ achievement of sustainable RTW, defined as reporting less than six weeks of working days missed out due to sickness absence within 12 months after first RTW. Secondary outcomes include health-related quality of life, mental functioning, RTW self-efficacy, overall job satisfaction, severity of mental illness and work ability. The health-economic evaluation will be conducted from a societal and public health care perspective, as well as from the employer’s perspective in a cost–benefit analysis. The design will be supplemented by a qualitative effect evaluation using pre- and post-interviews, and a multimethod process evaluation examining various predefined key process indicators from different stakeholder perspectives.

**Discussion:**

By applying a comprehensive, multimethodological evaluation design, this study captures various facets of RTW-PIA. In case of promising results for sustainable RTW, RTW-PIA may be integrated into standard care within German psychiatric outpatient clinics.

**Trial registration:**

The study was prospectively registered with the German Clinical Trials Register (DRKS00026232, 1 September 2021).

**Supplementary Information:**

The online version contains supplementary material available at 10.1186/s12888-023-04753-5.

## Background

Mental disorders (MDs) are highly prevalent worldwide [1, 2] and account for a large and increasing part of workforce sickness absence (SA) and disability in Europe [3]. It is estimated that 16% of the total global disability-adjusted life-years could be attributable to MDs [4], resulting in large economic costs to those affected, their employers and society at large [5, 6]. In Europe alone, the economic impact of MDs is predicted to make up more than 4% of its gross domestic product, equivalent to over €600 billion per year [7]. With average SA durations lasting more than twice as long as for all other diseases, MDs are known to be the second most common cause for SA in Germany, where the current study takes place [8]. Between 2000 and 2020, the proportion of mental health-related disability benefits has steadily and significantly increased in Germany, namely from 24.2% to 41.5% [9]. These figures are deeply concerning and call for greater prioritisation and investment for global mental health [10].

To facilitate the recovery process and prevent long-term negative consequences such as unemployment or disability pension [11–13], the return to work (RTW) of individuals with MDs is key [3, 14]. This is a complex process taking place at the intersection of the mental health care system and the workplace, in which the priorities of multiple involved stakeholders are not always aligned [15–17]. However, being back at work carries no guarantee of sustainable reintegration [18], given that most MDs are likely to relapse or recur easily [19, 20]. High rates of recurrent SA or multiple RTW attempts are commonly reported after an episode of mental health-related absence [21–23]. This underlines the necessity for designing both the overall treatment phase and RTW process as sustainably as possible in order to prevent relapses among employees with MDs and to minimise substantial future economic costs for society as a whole.

Despite an increasing body of research reported by existing systematic reviews [24–30], there is not yet conclusive evidence about the most effective RTW interventions on employment-related outcomes for individuals with MDs. In a systematic review by Nieuwenhuijsen and colleagues [27], specific work-directed interventions were not found to reduce the number of SA days in depressed employees compared to usual work-directed care alone. However, work-directed interventions (e.g. work modification, problem solving) combined with a clinical programme, such as psychological treatment, may have the potential to reduce the SA days on average by 25 days over one year compared to those receiving care as usual (CAU) only [27]. Another systematic review [26] demonstrated that RTW interventions involving elements of cognitive behavioural therapy significantly reduced the SA duration until RTW by approximately 13 days among individuals with a common mental disorder (CMD). Compared to CAU, these interventions did not yield significant differences regarding the overall success of RTW.

Mikkelsen and Rosholm [28] found a significant, but relatively small, positive effect of employment related interventions aimed at enhancing RTW for sick-listed employees with MDs. By conducting further meta-regression analyses, they demonstrated that interventions incorporating specific characteristics, such as workplace contact before RTW, gradual RTW as well as the use of more than one component, were more likely to be effective [28]. When looking at health-economic outcomes, interventions focusing on teaching employees coping skills (e.g. problem solving) for their RTW were found to be cost-effective concerning SA related to MDs [31].

### Sustainable RTW

Since reduced work ability and extended recurrent SA due to CMDs commonly persist after RTW [22], future research should address the sustainability of RTW after a period of SA by examining its long-term effects [15, 32, 33]. However, interventions targeting this sustainability and respective evaluation studies are scarce to date, partly because of the variation in definitions and operationalisations of sustainable return to work (SRTW). Knowledge about the numerous factors facilitating a SRTW after SA is therefore still limited [18, 34].

Nielsen et al. [18] proposed a preliminary definition of SRTW by including components such as returning to contracted working hours with equal earnings, minimal recurrence of long-term SA, functioning well at work, and not exiting prematurely from the labour market through long-term work disability or early retirement. Previous studies that examined SRTW among individuals with CMDs or musculoskeletal disorders have applied varying periods ranging from 30 days to seven months [34–37], in which returned employees stay at work with either zero or a pre-defined number of recorded relapses or SA recurrences. In contrast to this and in line with the German legislation, we have defined SRTW when employees report less than six weeks in total of contractually agreed working days missed out due to SA within 12 months after first returning to work.

### German mental health care system

Within the legal context of Germany, which is based on a social insurance system that focuses on protecting income [38], RTW-related efforts (e.g. gradual RTW [39]) have been ad hoc and not structurally supported across the various interfaces [40]. In a conservative welfare state regime like Germany [41], the national health care system is instituted on dichotomous principles which rest on different statutory, management and funding structures: medical vs. social services; hospital vs. ambulatory care; rehabilitation vs. acute treatment [42]. Since RTW is seen as a developmental process that begins with the onset of treatment, the topic of work or RTW-related components should be an integral part of therapy [16, 43, 44]. This continues to represent a culture change within German psychotherapeutic care, however [45].

To date, therapeutic efforts to get people with MDs into the labour market or keep them in the workplace have primarily taken place in the traditional vocational rehabilitation sector and thus outside the acute psychiatric treatment context [46]. With respect to the latter, psychiatric outpatient clinics (POCs), situated within general hospitals and networked with psychiatric hospitals and departments [42], are considered to be a cornerstone of psychiatric care in Germany [47]. Their multidisciplinary care approaches are aimed at patients who are not adequately reached by other contract medical care services within the mental health care system, especially in emergencies or after discharge [48]. Unlike English-speaking or Scandinavian countries, the labels outpatient clinics and community mental health teams are used interchangeably in Germany [42].

The awareness for implementing and evaluating well-known vocational rehabilitation program models, such as Individual Placement and Support [49], within the setting of POCs has recently been acknowledged [50]. These interventions focus on long-term unemployed patients with severe MDs to find non-demanding, supported employment. In contrast to this, we argue that treatment offered within the POCs needs to be supplemented by innovative work-directed aftercare interventions aimed at patients with employment contracts who wish to RTW after a period of MD-related SA.

### Aftercare interventions

Returning to work after post-discharge from psychiatric treatment remains fearful for many individuals with MDs [40]. They face various challenges regarding the transfer of acquired knowledge and behaviour changes but may also be confronted with individual, social, or work-related difficulties [51, 52]. The care of those affected should be seen less as a selective treatment measure and more as a continuous care process [51, 53]. In the field of tertiary prevention of chronic or recurrent MDs, various aftercare and follow-up interventions can therefore play an essential role in order to maintain the patient´s treatment gains and to prevent relapses [51, 53].

Aftercare interventions, which can be delivered in different forms and intensities, such as via monthly group meetings or web- and mobile technologies [51], could serve as a potential (blended-)care supplement for providing RTW assistance to patients who had already extended SA periods due to MDs. While supporting and accompanying a patient´s RTW process, aftercare treatment providers would have the chance to respond to problems arising in a timely and professional manner. However, such work-directed treatments for MDs are not yet a regular part of conventional aftercare programs in Germany. Since most aftercare concepts are provided in German outpatient rehabilitation centres [54–56], the implementation of timely and accessible aftercare programs focusing on SRTW among employees with MDs could additionally be a feasible option within POCs. POCs are normally located nearby the patients´ homes and workplaces and thus may allow access to work and clinical care during the RTW process.

### Development of a multimodal, clinical and work-directed intervention

RTW interventions that combine medical-psychotherapeutic approaches with work-directed components will be of substantial relevance for the further development of SRTW-practices. For this reason, an 18-month intensified RTW aftercare intervention, called **RTW-PIA** (German: Intensivierte **R**eturn **t**o **W**ork Nachsorge in **p**sychiatrischen **I**nstituts**a**mbulanzen von Versorgungskliniken), was developed to facilitate SRTW among sick-listed employees with MDs. It was established by multidisciplinary teams (e.g. psychotherapists, psychiatrists, occupational therapists, social workers), based on previous exploratory work carried out by RTW-experts which included the perspective of patients concerned [16].

RTW-PIA consists of the following three core modules which systematically build on each other throughout the clinical treatment and RTW process: face-to-face individual RTW support, monthly RTW aftercare group meetings and web-based aftercare. The modules aim to strengthen the participant’s work ability and RTW self-efficacy, to promote quality of life, functioning and job satisfaction and to reduce symptom severity. Each RTW-PIA module will be delivered by multidisciplinary psychotherapeutic treatment providers employed by POCs, who have received training for navigating participants through different RTW trajectories in a sustainable way.

The theoretical basis of RTW-PIA is rooted in the Conservation of Resources Theory [57], which postulates the need for resources in work life to accomplish future tasks and goals. RTW-PIA includes various approaches based on cognitive behavioural principles, mentalisation-based treatment, systemic therapy, or psychoeducation on occupational safety and health. To convey the mutual connections between illness and work ability, these approaches were enriched with work-directed components, such as the ’four-phase model of vocational reintegration’ [16]. This model provides a clearly structured process for a SRTW and is organised according to the suggested RTW-phases (co-orientation, coordination, cooperation, and re-co-orientation). In the context of the delivery of an individual RTW-support, these phases serve as a guideline for the treatment providers to discuss, prepare and follow-up the process of RTW in consultation with the participant. It has not yet been implemented in a psychiatric-psychosomatic system but is currently being tested in a workplace as a RTW intervention for individuals with symptoms of CMDs [58].

In addition, a framework which bundles and organises different work domains on various levels is the integrated ’IGLOO model’ [18] that promotes SRTW by considering the employee´s (post-)RTW resources in order to prevent future relapses. The model forms the basis of the RTW aftercare group meetings by strengthening the returnees´ resources at work across the **I**ndividual, **G**roup, **L**eader, **O**rganisational, and **O**verarching context level (IGLOO). The social support in the monthly group meetings aims to bolster the employees´ self-efficacy and self-confidence over the course of their return. To our knowledge, the transfer of the IGLOO model has not yet been tested in the clinical and RTW context in Germany.

Finally, in the course of the group meetings, we complement RTW-PIA with an individualised web-based aftercare, which is delivered in the sense of a blended- and stepped care approach. This component includes individual stress management, resilience training and mindfulness practices to ensure the future continuity of long-term self-care and proactive crisis management among employees.

### Evaluation design

To adequately evaluate RTW-PIA with its unique scope, a comprehensive, multimethodological evaluation design is necessary, consisting of an effectiveness, a health-economic and a process evaluation. Data triangulation using various quantitative and qualitative methods as well as several data sources will furthermore add to the extensive evaluation. Applying a long-term observation period of two years will help us to evaluate the sustainability of the employees´ RTW.

While a randomised controlled trial (RCT) is the most rigorous way to measure effectiveness [59], we argue that its usefulness can be highly improved when applied in combination with other evaluation instruments. Within the general field of RTW and mental health care, this is particularly true for economic evaluations since the take-up of RTW interventions into standard care is influenced by the results derived from cost-effect analyses [60, 61]. According to a recent review [31], the use of economic evaluations of RTW interventions is in its early phase, with only a few considering follow-up periods of more than 12 months [60]. However, quantitative evaluation methods alone cannot capture ‘how’ different key stakeholders appreciate and experience an intervention [62]. Data source triangulation is therefore recommended to gain multiple perspectives and identify the complexity and variety of some of the phenomena under study [63, 64].

Since effectiveness partially depends on how well the intervention was implemented, process evaluations can support better understanding of the underlying mechanisms [65, 66]. Integrating these above-mentioned approaches to evaluate RTW interventions is still underrepresented in the literature, however. To our knowledge, our comprehensive, multimethodological evaluation design taking place at the intersection of the mental health care systems and workplaces to examine SRTW among employees with MDs is the first of its kind in Germany.

### Aim and research questions

The overall aim is to present the design of a two-arm, multicentre, RCT to test the (cost-)effectiveness and implementation process of an intervention (RTW-PIA) aimed at sick-listed employees with MDs to achieve SRTW. The research questions (RQ) that we will address are:

- **RQ I** What is the effectiveness of RTW-PIA in conjunction with CAU on the achievement of SRTW in comparison with CAU only?
- **RQ II** Is RTW-PIA in conjunction with CAU superior in terms of cost-effectiveness and quality-adjusted life years gained compared to CAU only?
- **RQ III **What do the employees experience as promoting or hindering factors for their RTW and how do they experience RTW-PIA in this context?
- **RQ IV** How and to what extent has RTW-PIA been implemented in the participating psychiatric outpatient clinics with respect to the predefined key process indicators?

## Methods

### Design

**Fig. 1:**
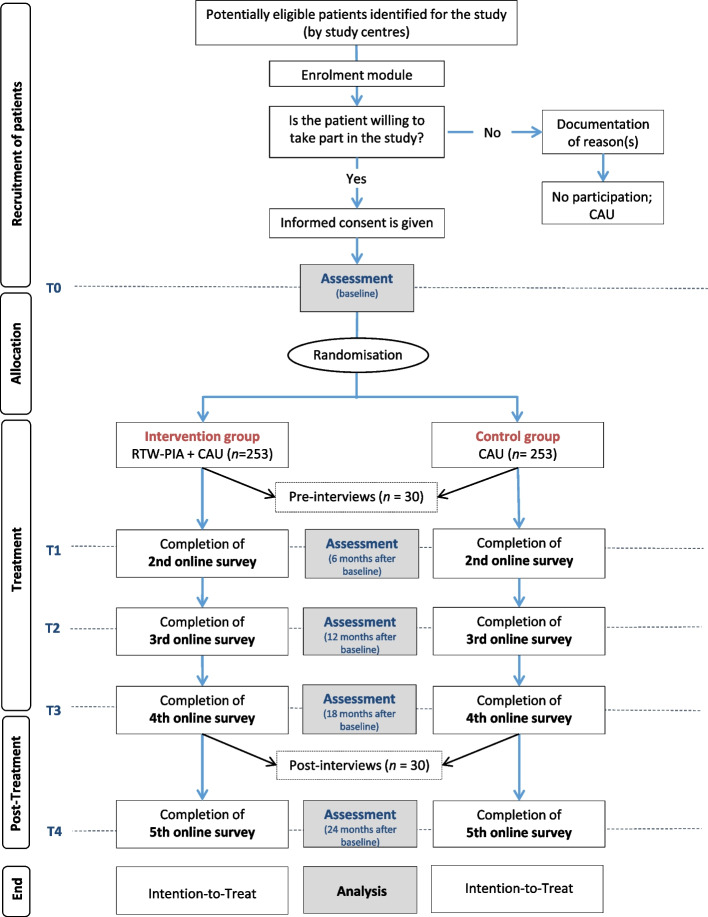
Flowchart of the recruitment procedure and study participants’ progress. Abbreviations: *T0*, Baseline; *T1*, 6 months -; *T2*, 12 months -; *T3*, 18 months -; *T4*, 24 months after completion of baseline survey; *CAU*, Care as Usual

This proposed study is designed as a multicentre RCT with one intervention group (IG) and one control group (CG). Participants will be randomly allocated (1:1) to either the IG and receive RTW-PIA in conjunction with CAU, or the CG and receive CAU only (Fig. 1). The study was approved by the Hanover Medical School, Germany, and prospectively registered at the German Clinical Trials Register on 1 September 2021. The present study protocol was compiled in accordance with the SPIRIT guidelines [67], and the final results will be published following the Consolidated Standards of Reporting Trials-Statement [68]. In this paper, we use the term “RTW-PIA” to refer to the name of the intervention to be evaluated.

### Study setting

The study will take place in a clinical setting in five German psychiatric outpatient clinics (POCs), situated within general hospitals and networked with psychiatric hospitals and departments. The POCs, selected ahead of the trial using convenience sampling, are distributed over four German federal states. Three of the POCs are located in major cities and the other two in medium-sized cities. All participating POCs belong to psychiatric communal hospitals, except for the POC located in Hanover, which is part of a university medical centre. Each study centre consists of a multidisciplinary treatment team (e.g. psychotherapists, social workers, psychiatrists, occupational therapists). All clinics are furthermore authorised for outpatient psychiatric-and psychotherapeutic care in accordance with § 118 of Volume V of the Social Insurance Code (German: Sozialgesetzbuch-Fünftes Buch) [69], as specified in the agreement between the National Association of Statutory Health Insurance Funds, the German Hospital Federation, and the Federal Association of Statutory Health Insurance Physicians [48].

### Interventions

Participants from the IG and CG will both receive access to CAU, i.e. the current therapeutic service standards that follows a needs-oriented approach and is delivered by a multidisciplinary team at the respective POC. Participants in the IG will furthermore receive RTW-PIA in conjunction with CAU. There are no restrictions with regard to concomitant care. Within each study centre, RTW-PIA will be delivered by separate teams that do not carry out CAU and who will therefore discuss treatment only in a case-based way.

#### CAU

CAU is customised according to the participant´s disorder, degree of severity, and associated impairments. Since the exposure to CAU can vary depending on the participant´s needs, the total duration of CAU was defined to last at least six months. The number of contacts in three months usually varies between 3 and 5, which can be 1 to 3 medical appointments, and 2 to 4 other appointments, e.g. social work consultations and appointments with a psychologist to coordinate non-medical treatment options (e.g. sports activities, ergotherapy, psychological counselling, and group therapy). Unlike RTW-PIA, CAU does not focus specifically on work, work ability, and RTW.

#### RTW-PIA in conjunction with CAU

RTW-PIA is an 18-month, multimodal, clinical and work-directed intervention that focuses on the individual, i.e. employees with MDs. RTW-PIA consists of three core modules (A-C) that systematically build on each other. Table 1 describes the RTW-PIA core modules in line with the Template for Intervention Description and Replication (TIDieR) checklist [70]. The modules will be organised and implemented by trained multidisciplinary psychotherapeutic treatment teams employed by the respective POCs. The exposure to each module may vary depending on the participant´s needs. IG-participants can receive up to 20 sessions in total (Module A: 3–8 individual sessions, Module B: 6–9 group meeting sessions, Module C: 1–3 video call sessions) besides CAU.

**Table 1:** Brief description of the RTW-PIA core modules according to the TIDieR checklist

*Brief name*	**Module A**. Individual RTW support	**Module B**. RTW aftercare group meetings	**Module C**. Web-based aftercare
*Why*	The aim is to provide professional support to the participants by assessing their current professional and personal obstacles and resources when returning to work. Helpful and hindering factors of the working environment will be assessed.	The aim is to develop confidence in the RTW process by strengthening the participant´s work ability and self-efficacy through mutual support, group cohesion and stigma reduction.	The aim is to facilitate a behaviour change during as well as after the RTW course so as to implement it in everyday work life. Participants should prepare themselves to deal proactively with inevitable crises and challenging working conditions so as to prevent future relapses.
*What (procedure)*	Individual support during the RTW-process is provided according to the four-phase model of vocational reintegration (*Co-orientation, Coordination, Cooperation, Re-co-orientation*) [16]. The process of RTW is discussed, prepared, and followed up in detail according to the phases. In some cases, the IG-TP may contact the company doctor (e.g. via phone) and accompany RTW meetings with the employer.	Aftercare group during the RTW process is provided according to the IGLOO model [18]. The treatment focuses on promoting resources, including individual resources (e.g. stress detection and management techniques, interpersonal skills at work), as well as on using the provided organisational and group-level resources to enhance working conditions. Mindfulness practices are provided at the start of every session via an e-mental health platform [71].	The web-based aftercare is provided on an e-mental health platform [71] and is aligned in concept and content with Modules A and B (e.g. stress management and mindfulness practices). It consists of 8 different thematic blocks. After the entry into a particular block that is coordinated by the IG-TP, participants can choose blocks according to personal preferences. This aftercare follows a blended-care approach, in which up to 3 video call sessions can be arranged.
*Who provides*	Each study centre consists of a multidisciplinary RTW-PIA treatment team (e.g. psychotherapists, psychiatrists, occupational therapists, social workers). The IG-TP should ensure continuity of treatment throughout Modules A-C.		
*How*	It consists of face-to-face individual sessions between the participant and IG-TP, and follows a needs-oriented approach. Thus, the delivered treatment by the IG-TP is less standardised.	It consists of monthly face-to-face group sessions, consisting of 3–9 people. IG-TP have a motivating, resource-oriented attitude. The treatment delivered is to some extent manualised and serves as orientation/guidance.	It combines therapy elements, free text journaling, video calls and psycho-educational information. At the end of each block, IG-TP will give asynchronous (written) feedback. Module C follows a needs-oriented approach for which a written procedure was jointly agreed.
*Where*	All sessions are delivered in the study centre, e.g. therapy rooms, and, if required, at the workplace of the participant.	Group therapy rooms in the study centres are available for the sessions.	It can be used on a PC/tablet/mobile phone from any desired location. Choosing a calm location is recommended.
*When and how much*	3–8 sessions (approx. 50 min/each) are scheduled for the first 6 months after study inclusion.	6–9 group meetings (100 min/each) are organised during the first 12 months after study inclusion.	It starts 6 months after study inclusion and can be used for 12 months. Usage time of 30 min/week is recommended.
*Tailoring*	Participation in 3–8 sessions is determined according to individual needs.	Participation in 6–9 sessions is determined according to individual needs, irrespective of the number of sessions of Module A attended.	It is provided to all IG participants, irrespective of the number of sessions of Modules A and B attended.
*How well planned*	All IG-TP have received at least one day of specific study training to ensure adherence to the intervention manual (see section of process evaluation, and Table 3 for further information). The IG-TP have biweekly case-based (cross-disciplinary) intervision which ensures good treatment quality.		

Abbreviation: *IG-TP* Intervention group-treatment providers

## Effectiveness evaluation

To test the effect of RTW-PIA, an effectiveness, a health-economic and a qualitative effect evaluation will be performed.

### Effectiveness and health-economic evaluation

In order to answer **RQ I** and **RQ II**, an effectiveness evaluation and a health-economic evaluation with a two-year observation period for each study participant will be designed and performed. The aim of the effectiveness evaluation is to investigate the effect of RTW-PIA in conjunction with CAU compared to CAU only with respect to the employees´ achievement of SRTW. The health-economic evaluation aims to assess the cost-effectiveness and cost-utility of RTW-PIA in conjunction with CAU compared to CAU only from a societal and public health care perspective, as well as the cost–benefit from the employer’s perspective within a time horizon of 6 months.

### *Study sample*

Eligible patients in this effectiveness study will meet the following inclusion criteria:

- aged between 18 and 60 years,
- employed for ≥ 15 h per week on the primary labour market^1^ according to German law,
- current sickness absence period of at least 6 weeks, based on self-report,
- current principal diagnosis of a mental disorder, classified according to the 10th version of the International Statistical classification of Diseases and Related Health Problems (ICD-10) from at least one of the following sections: schizophrenia, schizotypal and delusional disorders (F2); mood (affective) disorders (F3); neurotic, stress-related and somatoform disorders (F4); behavioural syndromes associated with physiological disturbances and physical factors (F5); disorders of adult personality and behaviour (F6), and
- fulfil all other entrance criteria for receiving treatment within the psychiatric outpatient clinics of psychiatric hospitals according to §118 (2) of Volume V of the Social Insurance Code [69].

Potential patients will not be eligible to participate if they ***a)*** have a current principal diagnosis of a mental disorder, classified according to the 10th version of the ICD-10, from one of the following sections: organic, including symptomatic, mental disorder (F0); mental and behavioural disorders due to psychoactive substance abuse (F1); ***b)*** have an acute and severe somatic disease (e.g. cancer); ***c)*** are currently pregnant; ***d)*** have decided against returning to the labour market; or ***e)*** have already planned and prepared an approved rehabilitation stay.

### *Recruitment procedure*

Patients who currently receive inpatient, day-care, or outpatient treatment in psychiatric clinics or POCs are screened for eligibility by the trained treatment teams of the respective study centres. A flowchart of the recruitment procedure and the study participants’ progress is shown in Fig. 1. Prior to study inclusion, all potentially eligible patients identified for the study will be invited to the one-time enrolment module “Work and Health”, which includes a psychoeducational group session on the following topics: interdependency of health and work, significance of work and its potential positive and negative effects, occupational integration management, and gradual RTW. At the end of the enrolment module, patients are informed about RTW-PIA and asked about their willingness to participate. Patients who give their written informed consent are then invited to the online baseline assessment. After successful completion of the baseline assessment, the patients are randomised to the IG or CG. The recruitment process started in September 2021.

### *Allocation and blinding*

Patients will be randomly assigned to either IG or CG with a 1:1 allocation, using permuted block randomisation with varying sizes stratified by study centre. Before recruitment began, five randomisation lists were prepared via an online randomisation service [72] by an independent researcher, blinded to the study conditions. For adequate allocation concealment, the generated lists were then securely transferred to an independent administrative working group, to which the evaluators, clinical practitioners, and participants never have access during recruitment. The baseline assessment takes place before group allocation to minimise reporting and selection bias. After completion, all participants will be randomised according to their chronological order of survey participation. The respective randomisation date will serve as the participant´s admission to the study. Blinding among study participants and treatment providers will not be possible before, during, or after the study. Due to the questionnaire design with items concerning the individual intervention modules, the evaluators will also not be blinded.

### *Data collection, outcomes and other measures*

The effectiveness and health-economic evaluation constitute the quantitative study part of the RCT. Self-reported quantitative data will be collected through online surveys and will be pseudonymised. Online assessments will take place at baseline (T0) and at 6 months (T1), 12 months (T2), 18 months (T3), and 24 months (T4) after completion date of the baseline assessment. All study participants will be regularly reminded to participate in the online surveys and receive compensation for their study-related efforts (€10 for each completed online survey). The overall study schedule of enrolment, interventions, instrument assessments as well as measurement time assessments is shown in Table 2. The primary outcome will be the achievement of SRTW. In addition, we will measure a range of secondary outcomes (e.g. health-related quality of life, mental functioning) as well as some further variables (e.g. to describe the sample). Since the study is addressed to German-speaking participants, translated questionnaires and/or ones validated for Germany were used in the online surveys. In the online surveys (T1-T4), the number of questions to be filled in depended on the respondent´s work status (meantime unemployed, etc.) and RTW status and situation (RTW event occurred, or not yet). A broad overview of all further measurements applied to the online survey can be found in Additional file 1.

**Table 2:**
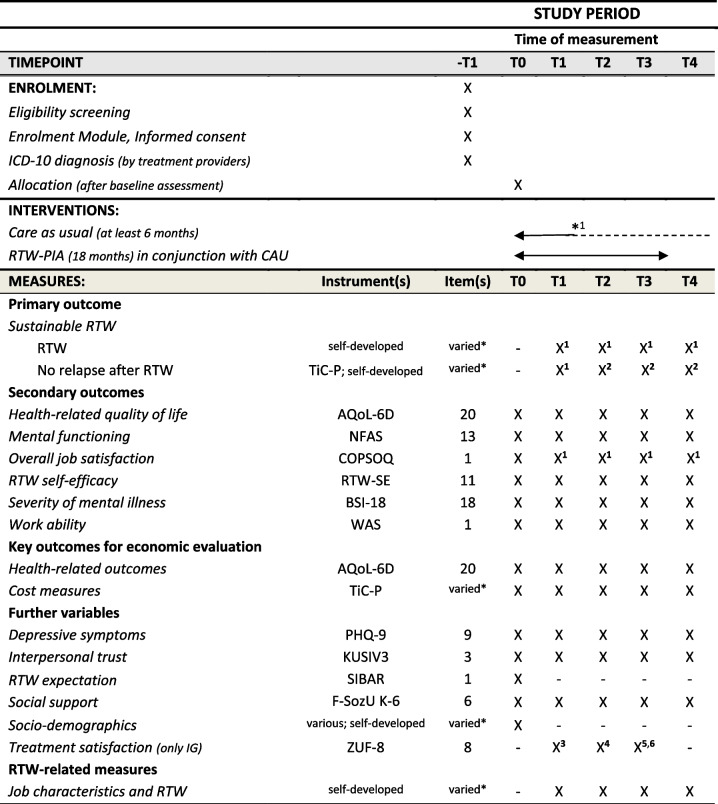
Study schedule of enrolment, interventions, and assessment

*-T1* Prior to study admission, *T0* Baseline, *T1* 6 months, *T2* 12 months, *T3* 18 months, *T4* 24 months after completion of baseline assessment. *AQoL-6D* Assessment of Quality of Life-6 Dimension [75], *BSI-18* Brief Symptom Inventory-18 [81], *COPSOQ* Copenhagen Psychosocial Questionnaire [78], *F-SozU K-6* Perceived Social Support Questionnaire brief form [91], *KUSIV3* Interpersonal Trust Short Scale [88], *NFAS* Norwegian Function Assessment Scale [76], *PHQ-9* Patient-Health Questionnaire-9 [87], *RTW-SE* Return to Work Self-Efficacy Scale [79], *SIBAR* Screening Instrument Work and Occupation [89], *TiC-P* Trimbos and Institute of Medical Technology Assessment Cost Questionnaire for Psychiatry [74], *WAS* Work Ability Score [83], *ZUF-8* Questionnaire for Patient Satisfaction [94]

^1^Only applied if participant did return to work

^2^Only applied once if participant´s first day back at work was more than 12 months ago

^3^Adapted for Module A

^4^Adapted for Module B

^5^Adapted for Module C

^6^Adapted for overall intervention

^*^Number of questions may vary depending on the participant´s response behaviour as well as the RTW-status

^*1^Duration of the intervention depends on the participant´s needs and may therefore vary

### Primary outcome

The primary outcome will be the employees´ achievement of sustainable RTW. We have defined SRTW when employees report less than six weeks in total of contractually agreed working days missed out due to sickness absence (SA) within 12 months after first returning to work. Thus, the measurement of the primary outcome is based on the following two items: **(I)** *RTW* and **(II)** *No relapse after RTW*. The self-adapted, single-item questions presented below are partially based on the Trimbos and Institute of Medical Technology Assessment Cost Questionnaire for Psychiatry (TiC-P) [73, 74].

#### (I) RTW

At T1, T2, T3 and T4, all employees will receive the following self-developed question: “*Have you meanwhile returned to work*?” with the answer options ‘*yes*’ and ‘*no*’. Meanwhile refers to the period since the last completed survey. The selected answer (yes) confirms the employee´s first RTW. The returned employees will be asked to enter their RTW date numerically (“*When was your first day back at work?*” (DD.MM.YYYY)).

#### (II) No relapse after RTW

Those employees who self-confirmed their RTW for the first time at either T1, T2, T3 or T4 (see (I)) will receive the following question: “*Did you stay home sick or call in sick for more than 6 weeks in total after your RTW?*” with the answer options ‘*yes*’ and ‘*no*’. The selected answer ‘*no*’ indicates that employees did not relapse after their RTW and might achieve SRTW in the future. Employees who self-confirmed their RTW to be more than 12 months ago at T2, T3, or T4 with “*My first day back at work was more than 12 months ago”* will receive the following question: “*In the first 12 months after your return to work, did you stay home sick or call in sick for more than 6 weeks in total?*” with the answer options ‘*yes*’ and ‘*no*’*.*

### Secondary outcomes for effectiveness evaluation

***Health-related quality of life ***will be measured with the Assessment of Quality of Life-6D (AQoL-6D) scale, which is composed of six domains (independent living, relationships, mental health, coping, pain, senses) [75]. The AQoL-6D has response scales that include 4–6 levels for each item, with higher scores indicating quality of life impairment. At baseline, the online survey included additional information for those still receiving inpatient treatment (“*For some questions, please imagine that you would have been at home if you are currently in the hospital*.”).

***Mental functioning*** will be measured with the 7-item Managing-scale and 6-item Cooperation/Communication scale from the Norwegian Function Assessment Scale (NFAS) [76]. The NFAS has a 5-point response scale, ranging from 1 (*no difficulty*) to 5 (*could not do it*). In accordance with [76], a response category item ‘*inapplicable to me, task not performed*’ was added and recorded as missing. The two NFAS subscale scores are each computed by calculating a mean score of the completed items, with low scores indicating good mental functioning [77].

***Overall job satisfaction*** will be measured with the following single-item question of the Job Satisfaction scale from the Copenhagen Psychosocial Questionnaire (COPSOQ) [78]: “*Regarding your work in general. How pleased are you with your job as a whole, everything taken into consideration?*” The single-item question has a 5-point response scale, with the answer options: *very satisfied*, *satisfied*, *unsatisfied*, *very unsatisfied*, and *not relevant*. However, we deleted the last response option and added ’*partly*’ as a third response option in between the scale to enable participants to respond neutrally to the question without forcing an answer in the positive or negative direction. The score of the single-item question ranges from 100 to 0, with higher scores indicating more job satisfaction.

***RTW self-efficacy*** will be assessed with the RTW self-efficacy scale (RTW-SE) [79], which was translated and verified in an earlier study [80]. The RTW-SE includes 11 items and has a 6-point response scale, ranging from 1 (*totally disagree*) to 6 (*totally agree*). A mean score over all 11 items is used to compute a total RTW-SE scale score, with higher scores indicating higher perceived self-efficacy regarding RTW.

***Severity of mental illness*** will be measured with three 6-item scales (somatisation, depression, anxiety) from the Brief Symptom Inventory-18 (BSI-18) [81]. The BSI-18 has a 5-point response scale, ranging from 0 (*not at all*) to 4 (*extremely*). The scores on each of the three BSI-18 scales range from 0 to 24. The sum score of the BSI-18, referred to as the Global Severity Index, ranges from 0 to 72, with higher scores indicating more distress [82].

***Work ability*** Will be measured with the single-item Work Ability Score (WAS) [83]. The WAS has an 11-point response scale, ranging from 0 (*completely unable to work*) to 10 (*lifetime´s best work ability*).

### Key outcomes for health-economic evaluation

#### Health-related outcomes

In the cost-effectiveness analysis, the main outcome will be the employees´ achievement of SRTW (yes/no). In the cost-utility analysis, quality-adjusted life years (QALYs), based on the AQoL-6D, will be the health-related outcome. The AQoL-6D generates patient preference-based utilities on a scale of 0 (death) to 1 (perfect health), using the time-trade-off method [84]. QALY gains will be estimated by calculating the area under the curve of linearly interpolated AQoL-6D utility values between assessment points to cover the follow-up period.

#### Cost measures

Costs will be measured from the societal, public health care and employer's perspectives. In the societal perspective, all costs related to the interventions (i.e. health care, patient and family and productivity costs) will be taken into account irrespective of who pays or benefits. Using the public health care perspective, only intervention and health care costs will be considered. When applying the employer's perspective, only costs and economic benefits pertinent to employers will be included (i.e. productivity costs) plus intervention costs, assuming that the latter would be paid for by the employer. For this, the TiC-P [73, 74], a retrospective questionnaire with a 3-month recall period and specifically adapted to patients of POCs, will be applied to collect data on health care use, patient and family costs, and productivity costs. Calculation of health care costs will be based on two German guidelines [85, 86].

### Further variables

The following variables will be predominantly used as possible outcomes, covariates, moderators, or mediators within further exploratory analyses.

***Depressive symptoms*** will be measured with the Patient Health Questionnaire (PHQ-9) [87]. The PHQ-9 has a 4-point response scale, ranging from 0 (*not at all)* to 3 (*nearly every day*). The sum score ranges from 0 to 27, with higher scores indicating the severity of depressive symptoms.

***Interpersonal trust*** will be measured with the Interpersonal Trust Short Scale (KUSIV3) [88]. The KUSIV3 has a 5-point response scale, ranging from 1 (*do not agree at all*) to 5 (*completely agree*). The sum score ranges from 3 to 15, with higher scores indicating higher interpersonal trust.

***RTW expectation*** will be assessed with a single-item from the Screening Instrument Work and Occupation (SIBAR) [89]. The question was slightly adapted with respect to the study-specific setting at baseline (*“Considering your current state of health, when do you think you will be able to return to work after your discharge?”*). The SIBAR has a 7-point response scale, with the answer options: *immediately*, *one month*, *three months*, *six months*, *nine months*, *one year or later*, and *not at all anymore*. As has been done before [90], the single SIBAR-item responses will be dichotomised into a’positive RTW expectation (≤ 3 months)’ and’negative RTW expectation (> 3 months)’.

***Social support*** will be measured with the brief version of the Perceived Social Support Questionnaire (F-SozU K-6) [91]. The F-SozU K-6 has a 5-point response scale, ranging from 1 (*not true at all*) to 5 (*very true*). The sum score ranges from 6 to 30, with higher scores indicating higher levels of general perceived social support.

***Socio-demographics*** will be assessed at baseline (T0) with several items including information on *gender*, *age, education, marital status, cohabitation, residence, total number of inhabitants of the place of residence* (extra item asking for the *number of inhabitants younger than 14 years*) [92], *occupational status, company sector, monthly gross household income* [73]*, degree of disability* [89]*, ethnicity, migration* [93] and *distance to the practice of the respective therapist or other practitioners* [73]. Data on *household income* will be collected at all five measurement points for the health-economic evaluation.

***Treatment satisfaction*** with the overall RTW-PIA treatment as well as with each RTW-PIA module (A-C) will be assessed with an adapted version of the Questionnaire for Patient Satisfaction (ZUF-8) [94]. The ZUF-8 has a 4-point response scale, ranging from 1 (*does not apply to me*) to 4 (*does totally apply to me*). The response category ‘*inapplicable to me, I did not participate’* was added to the scale to prevent socially desirable answers; scoring this answering category means that data are missing for the reason that the persons did not participate. In addition, the questions were slightly adapted with respect to the specific module (e.g. “*Please indicate the extent to which the following statements about Module B apply to you*”; (1) “*The group discussions I have participated in have so far been of high quality*”). As has been done before, the questions were rephrased as statements in order to have constant response categories within the ZUF-8 scale [95]. The sum score of the ZUF-8 ranges from 8 to 32 points, with higher scores indicating more treatment satisfaction.

### Job characteristics and RTW-related measures

Throughout the five online assessments (T0-T4), participants will be asked to answer questions related to job characteristics (self-developed and [96, 97]) so as to continuously capture their current working status (*contractually agreed weekly work hours and working days/week, thoughts of workplace shift, actual (internal) workplace shift, job loss experience,* etc.) and to assess possible adaptions/changes with regard to their unique employment contract details. Further information will be assessed with regard to RTW (e.g. *RTW to previous business (yes/no)*; *RTW to previous workplace (yes/no)* (if not applicable, *reason for company change* and *date*)), gradual RTW and the workplace integration management process. The paper by Sikora et al. [80] served as orientation for designing the questions.

#### Sample size and power calculation

In order to demonstrate a 15% increase in the proportion of employees from the IG achieving SRTW, based on [80] (CAU: 60%; RTW-PIA: 75%; power: 80%; significance level: 5%), the required sample size will be a minimum of 152 study participants per study arm. Calculations were performed with a sample size software [98]. Following previous authors [58, 99], we assumed a drop-out rate of 40%; thus, 506 participants will be needed in total.

#### *Statistical analyses*

### Effectiveness evaluation

The primary outcome will be analysed using a logistic regression model to determine the odds ratio of the intervention effect. First, we will estimate a baseline model including treatment group (IG vs. CG) as a dummy-coded predictor variable and SRTW achievement as the outcome variable. Second, we will compute an adjusted estimate by including the stratification variable of the study centres as well as, if necessary, other socio-demographic variables for which differences between IG and CG occurred despite randomisation. Weighted effect coding will be considered for study centre, using the largest group as the reference group. Third, we will explore interaction effects between treatment group and all baseline assessments of the secondary outcomes and further variables. In case of statistically significant interaction effects, we will report intervention effects for different values of the covariates. In these and subsequent models, we will adopt a significance level of 5% for testing the focal parameters and report corresponding 95% confidence intervals (CIs).

In the context of the secondary analysis, multiple-group latent growth curve models (LGCM) will be estimated to investigate the effect of the intervention on changes in health-related quality of life, mental functioning, overall job satisfaction, severity of mental illness and work ability. LGCM are based on a structural equation modelling framework and allow for a more flexible adaptation to the specifics of the data structure than many multilevel models [100]. For each secondary outcome variable, we will include data at five time points and estimate a multiple group latent-basis model. This model is a less restrictive variant of the linear growth curve model, i.e. it contains a latent intercept for baseline differences and a latent slope for change differences, but can also represent nonlinear forms of change. If necessary, we will allow the residual variances to differ between treatment groups and time points. The focal parameter in each model is the mean difference of the latent slopes between treatment groups.

In addition, we will explore several baseline variables as prognostic and prescriptive predictors of change. To do this, we will use the EffectLiteR approach and consider baseline variables in the multiple-group LGCM [101]. The focal test for potential prescriptive variables is an omnibus test for interactions between treatment group and baseline variables. All model parameters are estimated using full information maximum likelihood. To account for possible deviations from a normal distribution in outcome variables, we use scaled test statistics and robust standard errors.

All data will be analysed according to the intention-to-treat principle. Missing data for the primary and secondary outcomes are assumed to be “missing at random” (i.e. missing at random conditional on information from all variables included in the respective model). To make this assumption plausible, we will first correlate all baseline variables with the missingness of data at later time points, and then include all baseline variables that predict missingness in the estimation of focal parameters. For the analysis of the primary outcome, we will implement this using multiple imputation by chained equations (MICE) [102]. For the analyses of the secondary outcomes, we will use an auxiliary variables approach [103].

A per-protocol approach for the following two pre-defined IG subgroups will be conducted as sensitivity analyses: (1) for those who had attended at least 3 out of 8 Module A sessions, 6 out of 9 Module B sessions, as well as 1 out of 3 video call session(s) within Module C; and (2) for those who have provided their data at all measurement time assessments (T0-T4). Another sensitivity analysis will be conducted to investigate different diagnostic patient subgroups (e.g. CMDs (F3, F4) vs. all others).

Further details of the statistical analyses will be determined in an analysis plan, which will be pre-registered via the Open Science Framework [104] before database closure. Additional quality assurance and monitoring by an independent statistical expert will be considered throughout the project period. All effectiveness evaluation analyses will be performed using the statistical platform R 4.1 or higher.

### Health-economic evaluation

Missing cost and effect data will be imputed, using MICE. Two multilevel models will be specified, one for costs and one for effects, which consider the hierarchical structure of the data [105]. For effects, normal-based 95% CIs will be estimated. For the costs, 95% CIs will be estimated using bias-corrected and accelerated bootstrapping with 2,000 replications [106]. For this, cluster bootstrapping will be used, which is recommended for resampling hierarchical data [107]. The incremental cost-effectiveness ratio will be estimated by calculating the difference in costs between treatment groups divided by the difference in effectiveness of the treatment groups. The joint uncertainty surrounding costs and effects will be summarised using cost-effectiveness planes and cost-effectiveness acceptability curves [108]. To test the robustness of the base-case findings, probabilistic sensitivity analyses will be done by changing several assumptions made in the base-case scenario (e.g. about unit costs and volumes). The health-economic evaluation will be conducted using Stata.

### Qualitative effect evaluation

In order to answer **RQ III**, a qualitative effect evaluation will be performed. Its aim is to describe which interventions the participants used and how they benefited from them, how the interventions influenced their experience and actions in the RTW process, and which typical trajectories can be reconstructed in this context. For this purpose, both the participant´s implicit and explicit knowledge will be recorded.

For the qualitative sub-sample, study participants potentially interested in being interviewed (*n* = 30) from different study centres will be selected according to the following characteristics from the above recruited study population:

- IG and CG: 20 interview participants from the IG; and 10 participants from the CG (2:1 ratio),
- Gender-specific differences and similarities: men and women will be interviewed in a 1:1 ratio per group,
- Main ICD-10 diagnosis: proportionally, the most frequent MDs out of the total study population will be selected,
- Different subjective RTW expectations, and
- Other: variety in educational qualifications, company sector and size will be considered. In the sense of theoretical sampling [109], it will be decided after the analysis of the first interviews, which further characteristics might be of interest for the RQs.

### *Data collection and analysis*

The study participants will be interviewed at two time points. The pre-interviews take place at the beginning, after completion of the baseline survey and allocation (T0), either via face-to-face on site at the study centres, or by telephone or video call. The post-interviews will be conducted after the intervention (T3, 18 months) by telephone or video call (see Fig. 1). The duration of each interview will average 60 to a maximum of 90 min. All study participants will receive compensations for their study related efforts (€20 for each qualitative interview conducted). All interviews will be digitally recorded and transcribed in a pseudonymised form. The semi-structured interviews will include a narrative-generating main part as well as a reflexive and theoretically guided evaluative part.

The following topics will be considered for the pre-interview: (a) conditions under which the current crisis of MDs arose, (b) coping strategies used to deal with the disorder, (c) possible fears, hopes, and other factors that promote and inhibit a SRTW, (d) work-relatedness of the treatment, and (e) expectations with regard to the intervention. In contrast, the following post-interview topics will be covered: (f) experience and actions during and after the (gradual) reintegration, (g) self-confidence, trust, social support by the company and workplace accommodation changes during the RTW process, (h) promoting and inhibiting factors during the return to work, (i) support offered by the RTW experts as well as the experiences with the interventions, and their evaluations.

The Documentary Method of Interpretation for interview analysis [110, 111] will be used to elicit explicit knowledge (e.g. conscious, communicable skills and competencies, opinions, evaluations, and assessments) and tacit knowledge (e.g. internalised skills, orientations and knowledge based on experience). The six analysis steps will be as follows: creation of a thematic course per interview, identification of the narratively dense passages, formulating interpretation, reflective interpretation, case description per person and case comparisons and type formation. All interpretations and analyses will be discussed regularly by the qualitative research team to reach consensus.

## Process evaluation

In order to answer **RQ IV**, a multimethod process evaluation will be performed. The purpose of the extensive process evaluation is to assess the contextual factors, the implementation process, the framework conditions of RTW-PIA as well as the experiences of the study participants from the IG and the IG-treatment providers. The process evaluation will be guided by the proposed indicators from the theoretical framework of Linnan and Steckler [112] which have been aligned with RTW-PIA´s study context and its three core modules (A-C) (Table 3).

**Table 3:** Process indicators, data collection method

**Process indicators**	**Level**	**Operationalisation**	**Data collection method**
***Fidelity*** Adherence to the intervention manual and intervention delivery of Module B	IG-TP	Statements (ratings, yes/no) Reasons Suggestions for manual improvement	Evaluation sheets (Module B) Focus groups
***Dose delivered*** The extent to which each Module´s component (A-C) of RTW-PIA was actually delivered according to the intervention plan	IG-TP, IG	Number, date, and duration of every module´s sessions	Participant-related documentation sheet Technical log file (Module C)
***Dose received***	IG	Dose-response	Participant-related documentation sheet Needs-oriented evaluation sheet (Module A)
***Reach*** Actual proportion of participation in each Module (A-C) of the RTW-PIA intervention	IG	Characteristics of SC Characteristics of study participants Drop-out and reasons	Participant-related documentation sheet
***Satisfaction I*** Study participant´s satisfaction with RTW-PIA Modules (A-C) and the overall intervention	IG, Qualitative sub-sample	Satisfaction rate Quality rate Experiences	Online surveys (T1-T3) Post-Interview Technical log file (Module C)
***Satisfaction II*** Treatment provider´s satisfaction with RTW-PIA implementation	IG-TP	Experiences	Focus groups
***Context I*** Contextual factors and history that have an impact on the effectiveness of the intervention	Qualitative sub-sample	Description of barriers Description of facilitators	Pre-Interview Post-Interview
***Context II*** Contextual factors and history that have an impact on the effectiveness of the intervention	IG-TP	Description of barriers Description of facilitators	Focus groups
***Recruitment*** Procedures used to approach and attract patients; number of invited patients; reasons for non-participation	SC	Description of approaches	Logbook Weekly recruitment sheet Focus groups Anonymous non-response questionnaires

Abbreviations: *IG* Intervention group, *IG-TP* Intervention group-treatment providers, *SC* Study centre. For further information regarding time of measurements (T1-T3), please see Flow Chart (Fig. 1) or study schedule of interventions and assessments (Table 2)

The study population of the quantitative process evaluation will equal the participants of the IG (*n* = 253) from the above recruited study population and the participants of the CG from the qualitative sub-sample (*n* = 10). A sub-sample of IG-treatment providers from different study centres will be included as well.

### *Data collection and analysis*

Quantitative data collection of the process evaluation will take place for each IG-study participant in a pseudonymised form throughout the 18-month RTW-PIA treatment period. An overview of the key process indicators and the multimethod data collection approach is provided in Table 3. The quantitative data will be analysed by means of descriptive statistics (e.g. frequencies, means, summed up ratings). The pre- and post-interviews in the qualitative evaluation will complement the results of the process evaluation. Influential contextual factors will be recorded, evaluated, and processed. Specific information regarding their experiences and satisfaction with RTW-PIA will be collected from the IG-participants in the post-interview (*Satisfaction I*, Table 3). Furthermore, three qualitative focus groups will be conducted during the intervention period with a sub-group of involved IG-treatment providers from each study centre (*Fidelity, Satisfaction II,* and *Context I*, Table 3). All focus group meetings will be digitally recorded, transcribed in a pseudonymised form, and subsequently analysed thematically via qualitative content analysis, e.g. Kuckartz [113].

### Ethical aspects

The study protocol has been ethically approved by the principal Ethics Committee at Hanover Medical School. All original documents will be treated confidentially in line with German Federal Data Protection Act. A comprehensive data protection concept has been approved by the data protection officer of the Hanover Medical School. The contact details of participants will be stored separately in the respective study centre in locked cabinets or on secured servers and are only accessible to authorised staff. The evaluation data will be pseudonymised and stored on secured servers within the evaluator´s settings and be accessed only by authorised persons. A unique study identifier will enable the study participant´s online survey-, qualitative- and administrative data to be linked.

Even if rare, negative effects of psychotherapy cannot be completely ruled out [114]. The multidisciplinary treatment teams from each study centre will inform the evaluators about any occurrence of spontaneously reported adverse events and other unintended effects of the trial; the evaluators will document this information. With regard to the completion of the online survey, only few complications are expected since it contains mostly validated and frequently used instruments. At the end of each online survey assessment, participants will be reminded and advised to contact their respective psychotherapeutic treatment provider if the state of their health deteriorates. No interim analyses of data will be required or planned within the study due to its primary endpoint. A data monitoring committee has not been set up in the present study.

## Discussion

This study protocol describes the design of a study to evaluate the (cost-)effectiveness and implementation process of a multimodal, clinical and work-directed intervention, called RTW-PIA. By combining face-to-face individual RTW support, RTW aftercare group meetings as well as a web-based aftercare, RTW-PIA aims to support sick-listed employees with MDs to achieve SRTW. Although a recent review has demonstrated that RTW interventions combining both workplace components and clinical interventions could possibly help depressed employees to RTW faster and reduce the duration of sickness absence [27], it remains unclear which type and combination of clinical and work-directed interventions are effective and what the underlying mechanisms for their effectivity are.

We hypothesise that employees who receive RTW-PIA in conjunction with CAU will more frequently achieve SRTW at 12 months after first having returned to work, compared to those exposed to CAU only. In terms of cost–benefit ratio, we expect that RTW-PIA in conjunction with CAU will be superior to CAU only, thus generating cost savings for social insurance funds, companies and society as a whole. With respect to the qualitative evaluation, we strive to provide an in-depth analysis of how and under what conditions employees (do not) benefit from RTW-PIA. Within the framework of the multimethod process evaluation, we anticipate that our predefined key indicators will have an impact on the implementation of RTW-PIA.

The present study has some important strengths. Applying a randomised controlled study design will be the most appropriate method when evaluating the (cost-)effectiveness of RTW-PIA on the employees´ achievement of SRTW. We will include patients with a wide range of MDs to reflect the contemporary clinical practice in Germany. The present study will make an important contribution to the international research field of RTW by implementing an observation period of two years, enabling to examine long-term effects such as the sustainability of the employees´ RTW. By collecting and combining a considerable amount of outcome data on the (cost-)effectiveness and implementation process from several stakeholder perspectives, this multimethodological evaluation design will capture various facets of RTW-PIA in practice and will add to the evidence base for the optimisation of SRTW practices. In addition to publishing our results in international research journals, a revised RTW-PIA manual will be provided for practical use as well as the amended web-based aftercare, which is closely linked to the manual content-wise. Finally, the generalisability of our results across Germany will be supported by the participation of different POCs with both urban and rural contexts, distributed over four German federal states.

Despite this extensive evaluation design, this study faces some methodological limitations. Since there is no central register-based data available on the employees’ SA in Germany, we have to deal with self-assessed data. To minimise possible recall bias, participants will be asked to record the days of SA consecutively in a list provided by the study centres. In addition, the study participants and treatment providers cannot be blinded to the group allocation due to the psychosocial nature of the RTW-PIA modules. Therefore, the risks of both performance bias and subject expectancy bias are difficult to prevent and will be considered when interpreting the final results. Due to the COVID-19 crisis and its aftermath, we expect some challenges with regard to the recruitment of study participants and implementation of RTW-PIA within the clinical setting of POCs due to lower occupancy rates of the clinics, staffing shortages and hygiene measures. However, any possible deviations from the protocol with regard to the recruitment or delivery of RTW-PIA will be continuously documented and considered in the final evaluation.

To conclude, this protocol outlines the comprehensive, multimethodological evaluation design of a multimodal, clinical and work-directed RTW-intervention. To the best of our knowledge, this evaluation study to examine SRTW among employees with MDs is the first of its kind. In case of promising results, RTW-PIA may be integrated into standard care within various German POCs. This, in turn, could be of significant public health and economic relevance, considering the high prevalence of MDs, their large burden of disease and economic impact on social security systems and labour markets [7].

This study goes beyond the process of return to work by emphasising sustainable RTW with a two-year observation period. It focuses not only on health-related but also on work-related outcomes such as participant’s work ability, RTW self-efficacy, and job satisfaction. Due to its long-term perspective and comprehensive evaluation design, this study can advance knowledge in the international research field of RTW. Our findings can moreover be essential for health policy makers and other experts in the field to further develop evaluation studies and interventions targeting SRTW practices at the intersection of the mental health care system and the workplace.

## Supplementary Information

**Additional file 1.**

**Additional file 2.**

## Data Availability

The current article describes a study protocol, and therefore data sharing is not applicable as no datasets have yet been generated or analysed. The study material of this study is available on request from the corresponding author.

## Abbreviations

AQoL-6D: Assessment of Quality of Life-6 Dimension
BSI-18: Brief Symptom Inventory-18
CAU: Care as Usual
CG: Control Group
CI: Confidence Intervals
CMD: Common Mental Disorder
COPSOQ: Copenhagen Psychosocial Questionnaire
F-SozU K-6: Perceived Social Support Questionnaire brief Form
ICD-10: International Statistical Classification of Diseases and Related Health Problems
IG: Intervention Group
IGLOO: Individual, Group, Leader, Organisational, and Overarching Context Level
IG-TP: Intervention group-treatment providers
KUSIV3: Interpersonal Trust Short Scale
LGCM: Latent Growth Curve Models
MD: Mental Disorder
MICE: Multiple Imputation by Chained Equations
NFAS: Norwegian Function Assessment Scale
PHQ: Patient Health Questionnaire
POC: Psychiatric outpatient clinics
QALY: Quality-Adjusted Life-Year
RCT: Randomised controlled trial
RQ: Research Question
RTW: Return to Work
RTW-PIA: Intensivierte Return to Work Nachsorge in psychiatrischen Institutsambulanzen von Versorgungskliniken
RTW-SE: Return to Work-Self Efficacy
SA: Sickness Absence
SC: Study Centre
SIBAR: Screening Instrument Work and Occupation
SRTW: Sustainable Return to Work
TiC-P: Trimbos and Institute of Medical Technology Assessment Cost Questionnaire for Psychiatry
TIDieR: Template for Intervention Description and Replication
WAS: Work Ability Score
ZUF-8: Questionnaire for Patient Satisfaction

## Footnotes

1 those employed at a public or private employer (thus salaried workers) without public subsidies supporting employment.
