# Supplementary material for: Evaluating a multimodal, clinical and work-directed intervention (RTW-PIA) to support sustainable return to work among employees with mental disorders: study protocol of a multicentre, randomised controlled trial
Source: BMC Psychiatry. 2023 May 30;23:380. doi: 10.1186/s12888-023-04753-5 (PMC10228446; doi:10.1186/s12888-023-04753-5)
Supplement: Supplementary file 1 — Additional file 1. [file 12888_2023_4753_MOESM1_ESM.pdf]

Supplementary material. Evaluating a multimodal, clinical and work-directed intervention (RTW-PIA) to support sustainable return to work among employees with mental disorders: study protocol of a multicentre, randomised controlled trial **| Appendix I.** Overview of further measurements applied to the online surveys

		Time of measurement				
**Job and RTW-related measures** (self-rated):	**Instrument**	**T0**	**T1**	**T2**	**T3**	**T4**
**RTW-specific**						
RTW to previous workplace	self-developed	-	X[1]	X[1]	X[1]	X[1]
RTW to previous business	self-developed	-	X[1]	X[1]	X[1]	X[1]
Total days of sickness absence days after RTW (numeric input)	TiC-P; self-developed	-	X[1]	X[1]	X[1]	X[1]
**Incapacity to work**						
Total number of sickness absence days (up to the date of baseline completion)	TiC-P; self-developed	X	-	-	-	-
Total days of sickness absence days within the last 12 month (numeric input)	TiC-P; self-developed	X	-	-	-	-
Total days of sickness absence days since the last survey (numeric input)	TiC-P; self-developed	-	X	X	X	X
**Work related**						
Contract of employment (limited / unlimited)	self-developed	X	-	-	-	-
Contractually agreed working days and - hours/week	self-developed	X	-	-	-	-
Management function and responsibility	self-developed	X	-	-	-	-
Company size	self-developed*	X	-	-	-	-
Change of workplace	self-developed	-	X	X	X	X
Changes of contractually agreed working days and - hours/week	self-developed	-	X	X	X	X
Perceived management´s concern for the workers' psychological health	PSC-4	X	-	-	-	-
Quantitative and workplace demands	COPSOQ	X	X[1]	X[1]	X[1]	X[1]
Physical demands	COPSOQ	X	X[1]	X[1]	X[1]	X[1]
Influence at work	COPSOQ	X	X[1]	X[1]	X[1]	X[1]
Support from colleagues	COPSOQ	X	X[1]	X[1]	X[1]	X[1]
Support from supervisor	COPSOQ	X	X[1]	X[1]	X[1]	X[1]
Supervisor change in the meantime	self-developed	-	X[1]	X[1]	X[1]	X[1]
Quality of leadership	COPSOQ	X	X[1]	X[1]	X[1]	X[1]
Work privacy conflict	COPSOQ	X	X[1]	X[1]	X[1]	X[1]
Insecurity at work	COPSOQ	X	X	X	X	X
Thoughts of jobsite change	COPSOQ	X	X	X	X	X
**Gradual Return to Work (GRTW) process** (Stufenweise Wiedereingliederung; STWE - Gradual RTW)						
Awareness of GRTW-Term and recommendation	self-developed	X	-	-	-	-
Consideration to accept GRTW	self-developed	X	X	X	X	X
GRTW-Rejection reasons (Multiple answers)	self-developed	X	X	X	X	X
Date of official GRTW start	self-developed	-	X	X	X	X
Current state of GRTW	self-developed	-	X[a]	X[a]	X[a]	X[a]
Period of time	self-developed	-	X[a]	X[a]	X[a]	X[a]
Possible conversation execution with employer before start of GRTW	self-developed	-	X[a]	X[a]	X[a]	X[a]
GRTW time schedule	self-developed	-	X[a]	X[a]	X[a]	X[a]
GRTW content planning	self-developed	-	X[a]	X[a]	X[a]	X[a]
Possible GRTW-feedback conversation execution (Multiple answers)	self-developed	-	X[a]	X[a]	X[a]	X[a]
Self-assessment of task distribution during GRTW (possible over-/underload)	self-developed	-	X[a]	X[a]	X[a]	X[a]
Duration of GRTW	self-developed	-	X[a]	X[a]	X[a]	X[a]
Overall self-rated helpfulness of GRTW	self-developed	-	X[a]	X[a]	X[a]	X[a]

T0, Baseline; T1, 6 months -; T2, 12 months -; T3, 18 months -; T4, 24 months after completion of baseline assessment. COPSOQ, Copenhagen Psychosocial Questionnaire; PSC-4, Psychosocial Safety Climate; TiC-P, Trimbos and Institute of Medical Technology Assessment Cost Questionnaire for Psychiatry. [1] Only applied if participant did return to work; [a] only applied if participant started a gradual RTW since the last survey. * in accordance with small and medium-sized enterprise EU-definition

Supplementary material. Evaluating a multimodal, clinical and work-directed intervention (RTW-PIA) to support sustainable return to work among employees with mental disorders: study protocol of a multicentre, randomised controlled trial **| Appendix I.** Overview of further measurements applied to the online surveys

		Time of measurement				
**Job and RTW-related measures** (self-rated):	**Instrument**	**T0**	**T1**	**T2**	**T3**	**T4**
**"RTW/BEM" process** (Betriebliches Eingliederungsmanagement; BEM - Workplace integration management process)						
Awareness of BEM-Term	self-developed	X	-	-	-	-
BEM - Availability at work	self-developed	X	-	-	-	-
Receipt of a BEM-offer	self-developed	X	X	X	X	X
Consideration to accept BEM	self-developed	X	-	-	-	-
BEM-Rejection reasons (Multiple answers)	self-developed	X	X[b]	X[b]	X[b]	X[b]
Date of BEM-offer	self-developed	-	X[b]	X[b]	X[b]	X[b]
First BEM-meeting	self-developed	-	X[b]	X[b]	X[b]	X[b]
Attendees/Conversation partners within BEM meeting (Multiple answers)	self-developed	-	X[b]	X[b]	X[b]	X[b]
BEM acceptance	self-developed	-	X[b]	X[b]	X[b]	X[b]
Possible implemented BEM measures (Multiple answers)	self-developed	-	X[c]	X[c]	X[c]	X[c]
Completion of BEM	self-developed	-	X[c]	X[c]	X[c]	X[c]
Overall self-rated helpfulness of BEM	self-developed	-	X[c]	X[c]	X[c]	X[c]
**RTW measures**						
Helpful/necessary work accommodation needs (Multiple answers)	self-developed	X	-	-	-	-
Main reasons for no RTW (yet) (Multiple answers)	self-developed	-	X	X	X	X
Further implemented measures (Multiple answers)	self-developed	-	X[1]	X[1]	X[1]	X[1]
**Other**						
First diagnosis	ICD-10	X	-	-	-	-
Cause of illness	Adapted from WS	X	-	-	-	-
Actual work situation (e.g. (early-)retirement, full pension, unemployment)	self-developed	-	X	X	X	X
Application for reduced earning capacity pension	Adapted from SIBAR	X	X	X	X	X
Application for partial disability pension	Adapted from SPE-Scale	-	X	X	X	X
Incivility	WIS	X	X[1]	X[1]	X[1]	X[1]
Perceived stress	PSS-10	X	X	X	X	X
Recovery	FABA	X	X[1]	X[1]	X[1]	X[1]
Self-related overall health	SOEP	X	X	X	X	X
Morbidity	WAI	X	-	-	-	-
Sickness presentism	TiC-P	X	X	X	X	X
Type of health insurance (private, statutory)	self-developed	X	-	-	-	-
**Treatment**						
Treatment (in-/outpatient, day clinic,…)	self-developed	X	-	-	-	-
Work-relatedness discussed during stay in the clinic (or treatment)	self-developed	X	-	-	-	-
Treatment (in-/outpatient)	self-developed	-	X	-	-	-
Clinic discharge	self-developed	-	X	-	-	-
Healthcare utilisation	TiC-P	X	X	X	X	X

T0, Baseline; T1, 6 months -; T2, 12 months -; T3, 18 months -; T4, 24 months after completion of baseline assessment. FABA, Questionnaire for the Analysis of Stress-relevant Coping with Demands (German: Fragebogen zur Analyse belastungsrelevanter Anforderungsbewältigung); SIBAR, Screening Instrument Work and Occupation (German: Screening-Instrument zur Messung des Bedarfs an berufsbezogenen Behandlungsangeboten in der medizinischen Rehabilitation); SOEP: The German Socio-Economic Panel Study; PSS: Perceived Stress Scale; SPE-Scale, Subjective Prognostic Employment Scale; TiC-P, Trimbos and Institute of Medical Technology Assessment Cost Questionnaire for Psychiatry; WAI: Work Ability Index; WIS, Workplace Incivility Scale; WS. Würzburger Screening. [1] Only applied if participant did return to work; [b] only applied if participant received an offer for an operational integration management since the last survey; [c] only applied if participant accepted the operational integration management offer.
